# A Dominant Gene for Male Sterility in *Salvia miltiorrhiza* Bunge

**DOI:** 10.1371/journal.pone.0050903

**Published:** 2012-11-27

**Authors:** Zhiming Shu, Zhen Wang, Xiaoqian Mu, Zongsuo Liang, Hongbo Guo

**Affiliations:** Shaanxi Research Center of TCM Fingerprinting and NP Library, College of Life Sciences, Northwest A&F University, Yangling, PR China; United States Department of Agriculture, United States of America

## Abstract

A natural male sterile mutant of *Salvia miltiorrhiza* (Labiatae, Sh-B) was found during field survey in 2002. Our objective was to analyze its genetic mechanism for producing F1 hybrid seeds and to develop a molecular marker linked to male sterile gene for selection of a hybrid parent line. The segregation ratios of male sterile plants to fertile plants in the progenies of both testcross and backcross were 1:1 in continuous experiments conducted in 2006–2009. The male sterile Sh-B was heterozygous (Msms). The male sterile plants could capture most pollen (2 granule/cm^2^·24 h) with row ratio (female : male 2 : 1) within 45-cm distance and harvest the largest amount of 6495 g hybrid seeds per hectare. We also developed DNA markers linked to the male sterile gene in a segregating population using bulked segregant analysis (BSA) and amplified fragment length polymorphism (AFLP) techniques. The segregating population was subjected to BSA-AFLP with 128 primer combinations. One out of fourteen AFLP markers (E11/M4208) was identified as tightly linked to the dominant male sterile gene with a recombination frequency of 6.85% and at a distance of 6.89 cM. This marker could be converted to PCR-based assay for large-scale selection of fertile plants in MAS (marker-assisted selection) at the seedling stage. Blastn analysis indicated that the male sterile gene sequence showed higher identity with nucleotides in *Arabidopsis* chromosome 1–5, and was more likely to encode S-adenosylmethionine-dependent methyltransferase, in which DNA methylation regulated the development of plant gametogenesis.

## Introduction

In the past several decades, tremendous economic benefits has been obtained worldwide by using male sterility as a genetic tool to control pollination and to produce hybrid F1 seeds in many crops [1]. However, hybrid medicinal plant cultivars have few reports and not been produced extensively in large-scale production of medicinal materials. To date, only three medicinal plant species with male sterility have been reported, *Salvia miltiorrhiza* Bunge [2], [3], *Platycodon grandiforus* (Jacq.) A. DC. [4], [5], [6], and *Lycium chinense* Miller [7].

The roots and rhizomes of *S*. *miltiorrhiza* have been used as traditional Chinese herb drug (known as Danshen) for removing blood stasis, alleviating pain, promoting the circulation of blood, promoting menstruation, tranquilizing the brain, and treating cardiovascular and cerebrovascular diseases [8], [9]. Due to increasing demand in recent years, its cultivation area is rapidly expanding and out-of-order introduction among regions is generally occurring, which makes seeds contamination and degradation more severely and thus resulting in inconstant quality of medicinal materials. Moreover, there is no hybrid cultivar till now for *S*. *miltiorrhiza*, which also is disadvantageous to quality control of medicinal materials even for those individuals growing in the same field.

Since a natural male sterile mutant of *S*. *miltiorrhiza* (Sh-B) was firstly found during our field survey in 2002, research has been conducted to determine its pollen development [2] and biological characteristics [3]. Amplified fragment length polymorphism (AFLP) technique, one of the most efficient molecular marker systems for screening genes of interest [10], [11], [12], is employed in this study to screen the markers that might link with the dominant male sterile gene.

Therefore, the present investigation is undertaken to determine: 1) the segregation in progenies of both backcrosses and testcross for producing F1 hybrid seeds; 2) screening AFLP markers that associate with male sterility gene in *S*. *miltiorrhiza*.

## Materials and Methods

### Plant Material

The male sterility of *S*. *miltiorrhiza* (Sh-B) was derived from natural mutants found in field survey in 2002 [3]. The segregating population (273 individuals) was constructed by consecutively backcrossing between Sh-B and fertile plants at a ratio of 1∶1 (fertile : sterile). An F_2_ population obtained from testcross between sterile and inbred fertile plants was used to practical test of the markers linked to the male sterile gene. Both male sterile and fertile plants were grown on the experimental farm of Northwest A & F University.

### Genetic Analysis and Field Production of F1 Hybrid Seeds

**Figure 1:**
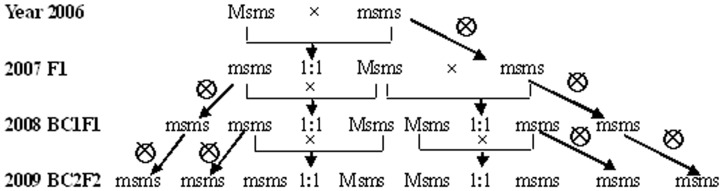
Genetic pattern of male sterility of *Salvia miltiorrhiza* deduced by segregation result in Table 1 through backcross and testcross.

Male sterile line Sh-B was crossed with three fertile lines (A-20, A-249, A-207) to determine the fertility of F1s and to observe F2 segregation for male sterile and fertile plants (Fig. 1, Table 1). The sterile F1 seeds (half of total) were backcrossed to self-pollination progenies of fertile lines to determine the BC1F1 segregation ratios of male-sterile and fertile plants. The BC1F1s were assessed by both testcross and backcross and BC2F2 segregation ratios of male-sterile and fertile plants were determined only in progenies of BC1F1 families that segregated for male sterile plants.

**Table 1:** Segregation of male fertile and sterile plants of *Salvia miltiorrhiza* (Sh-B) in backcross and testcross populations tested over three years.

Cross*	Year	No. of plants observed		Segregation ratio	Probability
		Fertile plants	Sterile plants		
(Sh-B×A-20) F1	2007	155	148	1:1	0.17
(Sh-B×A-249)F1	2007	174	169	1:1	0.22
(Sh-B×A-107)F1	2007	207	193	1:1	0.13
(Sh-B×A-20) BC1F1	2008	206	195	1:1	0.21
(Sh-B×A-249)BC1F1	2008	193	189	1:1	0.29
(Sh-B×A-107)BC1F1	2008	205	198	1:1	0.18
(Sh-B×A-20) BC2F2	2009	206	200	1:1	0.15
(Sh-B×A-249)BC2F2	2009	194	185	1:1	0.22
(Sh-B×A-107)BC2F2	2009	195	193	1:1	0.62
Sh-B×A-20Sh-BA	2009	215	211	1:1	0.22
Sh-B×A-107Sh-BA	2009	209	202	1:1	0.18
Sh-B×A-249Sh-BA	2009	207	199	1:1	0.15
A-20^ 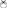 ^	2008	120	0		
A-249^ 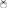 ^	2008	140	0		
A-107^ 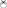 ^	2008	150	0		
A-20Sh-BA^ 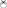 ^	2008	180	0		
A-107Sh-BA^ 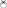 ^	2008	170	0		
A-249Sh-BA^ 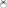 ^	2008	190	0		

* A-20, A-249 and A-107 were sterile and inbred lines of *Salvia miltiorrhiza* with different biological characteristics; BC1F1 represented F1 progeny with one backcross; BC2F2 represented F2 progeny with twice backcross; ^
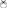
^represented self pollination; A-20Sh-BA represented half of fertile plants obtained from sterile seeds after hybridization between A-20 × Sh-B; A-20Sh-BA was self pollination of A-20Sh-BA progeny.

Given that half of F1 plants were fertile, identified till to flowering and they were removed from the field before harvest. To obtain more seedlings with uniform male sterility used to produce F1 hybrid seeds, the asexual reproduction method by using root cuttings as ‘seeds’ for sterile plants was chosen due to its higher propagation index (general 4–5 root cuttings/one root) than that of stem base cuttings (general 2–3 cuttings/one base).

To obtain the largest yield of F1 hybrid seeds from male sterile plants, four row ratios (female: male = 2∶1, 2∶2, 3∶2, 4∶4) were investigated. Each row ratio field experiment was conducted in an isolated region to avoid pollen contamination each other. Pollen flow was also observed at full-flowering stage to determine its spread distance (30, 45, 60 and 75 cm were estimated), corresponding with different row ratios. Pollen was captured by sticky microscope slides (length 76.2, width 25.4 and thickness 1.1 mm) coating with vaseline, which were placing on tripods with the same height as flower from last afternoon 6:00 PM to the next afternoon 6:00 PM. The number of pollen grains was then calculated under microscope within the range of all slide surface. In addition, both seed setting rate (percentage of seeding flowers/all flowers) and seed index (the number of seeds/all flowers), as well as the yield of F1 hybrid seeds were all calculated.

### DNA Extraction and AFLP Analysis

Fresh young leaves of *S*. *miltiorrhiza* from each plant were collected and DNA was extracted by using an improved cetyltrimethylammonium bromide (CTAB) method [13]. To screen AFLP markers linking to male sterile gene, equivalent amounts of DNA from eight randomly selected sterile individuals were pooled to construct sterile bulk and another eight fertile individuals for fertile bulk. The AFLP procedure followed was the one described by Vos et al. [14].

**Table 2:** Pollen flow and seed set of male sterile plants of *Salvia miltiorrhiza* when producing hybrid F1 seeds with different row ratios.

Row ratio female:male	Pollen flow at distance (cm) (gradule/24 h)					Seed setting rate (%)	Seed index	Seed yield per individual (g)	Seed yield per hectare (g)
	30	45	60	75	Average				
2:1	2	2			2	54.7	0.30	0.50	6495
2:2	2	2			2	54.2	0.29	0.32	3120
3:2	2	2	1		1.67	47.6	0.27	0.49	5730
4:4	2	2	1	1	1.5	44.0	0.23	0.42	4095

### AFLP Fragment Cloning and Sequencing

Bands containing targeted AFLP fragments were exercised from polyacrylamide gel and then were placed in a 0.5 ml eppendorf tube. The gel slices were crushed with a pipette tip and boiled at 100°C for 15 minutes after adding 50 µl of double-distilled water. The tubes were centrifuged at a velocity of 10,000 rotation per minute for 5 minutes. 5 µl of the supernatant was used as template solution for selective amplification with the same primer combination. The re-amplified PCR product was analyzed with a 1.2% agarose gel. A UNIQ-10 EZ Spin Column DNA Gel Extraction Kit (Sangon, Shanghai) and a pGEM-T Easy Vector (Tiangen, Beijing) were used to purify and clone the polymorphism fragments. Then white colonies growing on Amp+/X-gal/IPTG LB solid media plates were selected and cultured overnight in LB liquid media. Colonies containing target fragments were sequenced by Sangon Biotech (Shanghai) Co. Ltd.

The homology of the sequenced AFLP markers linked to male sterile gene was determined using BLASTn by comparison with database at NCBI (http://www.ncbi.nlm.nih.gov/BLAST).

### Linkage Analysis

The polymorphic AFLP markers were identified in the complete F1 mapping population only those exhibiting reproducible polymorphisms between fertile DNA bulks and sterile DNA bulks. The fertility and molecular marker data were combined for linkage analysis using the software package MAPMAKER/EXP 3.0 [15], [16]. The recombinant frequencies between male sterile gene and AFLP markers were calculated through two-point tests and linkage map was constructed by three-point or multiple-point tests with a minimum LOD threshold of 3.0. The recombination values were converted into centiMorgans (cM) by using Kosambi mapping function [17].

**Figure 2:**
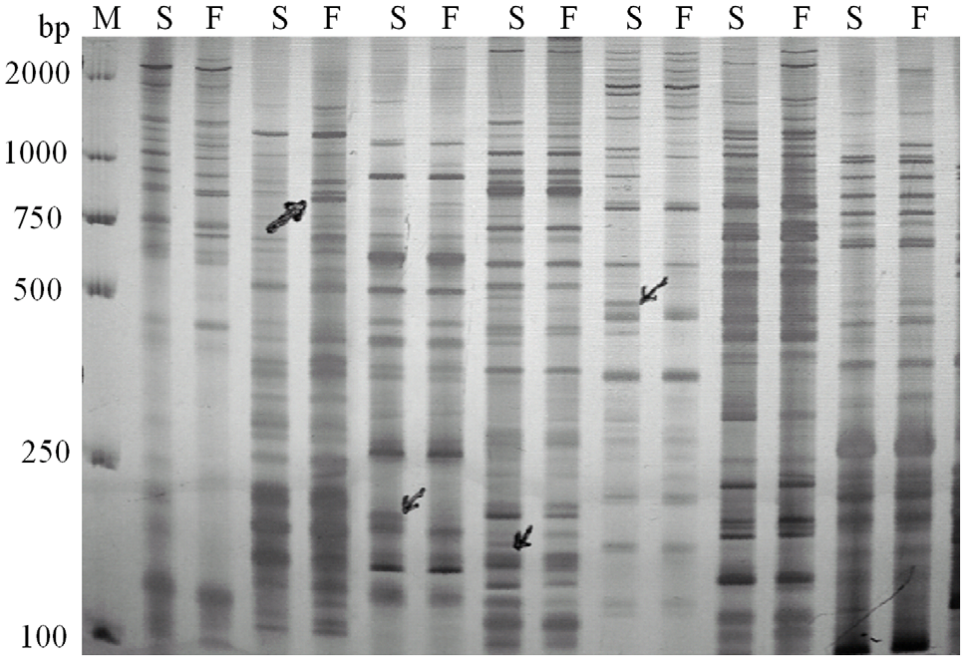
Selective amplification in male sterile (S) and fertile (F) populations of *Salvia miltiorrhiza* by AFLP. Arrows represented differently expressed bands between them.

**Figure 3:**
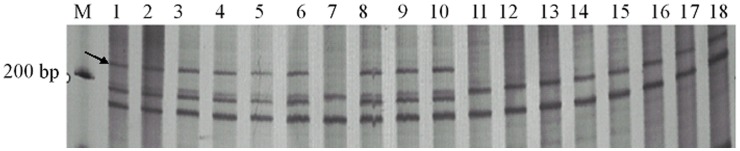
AFLP amplification profiles in male sterile and fertile plant populations generated by primer combination E11/M4_208_. Lanes: 1–9 sterile individuals; 10–18 fertile individuals; The arrow represented the band that tightly linked with dominant male sterile gene Of *Salvia miltiorrhiza.*

## Results and Discussion

### Segregation Analysis

The segregation of male sterility of both testcross and backcross progenies were all showed a 1∶1 ratio suggesting a dominant control (Ms) (Fig. 1, Table 1). The male sterile Sh-B was heterozygous (Msms). Given that all male sterile plants (Msms) were stable sterile, it was impossible to perform self-pollination and to obtain homozygous male sterile plants (MsMs).

**Table 3:** Blast hits of AFLP fragment E11/M4208 that tightly linked with male sterile gene in *Salvia miltiorrhiza*.

GenBank Acc. No.	Blast hits	Organism	E - value	Identities (%)
NC003071.7	Hypothetical protein, 2640 bp at 5′ side: ethanolaminephosphotransferase; 314 bp at 3′ side: MADS-box protein	*Arabidopsis thaliana*, chromosome 2	0.12	93
NC003076.8	zeaxanthin epoxidase (ZEP) (ABA1)	*Arabidopsis thaliana*, chromosome 5	5.2	95
NC003075.7	dicarboxylate carrier 2	*Arabidopsis thaliana*, chromosome 4	5.2	91
NC003074.8	hypothetical protein	*Arabidopsis thaliana*, chromosome 3	5.2	84
NC003070.9	S-adenosylmethionine-dependent methyltransferase domain-containing protein	*Arabidopsis thaliana*, chromosome 1	5.2	100
NC008401.2	5016 bp at 5′ side: Hypothetical protein; 256 bp at 3′ side: Hypothetical protein	Oryza sativa Japonica Group DNA, chromosome 8	1.4	82
NC008398.2	38938 bp at 5′ side: Hypothetical protein; 11051 bp at 3′ side: Hypothetical protein	Oryza sativa Japonica Group DNA, chromosome 5	1.4	92
NC008400.2	Hypothetical protein	Oryza sativa Japonica Group DNA, chromosome 7	4.8	100

### F1 Hybrid Seeds Production

The male sterile line (as female) and a fertile inbred line (as male) were planted together with four row ratios (2∶1, 2∶2, 3∶2, 4∶4) in each isolation area for production of F1 hybrid seeds (Table 2). The male sterile plants could capture most pollen flow (2 pollen granule/cm^2^·24 h) with row ratio (female:male 2∶1) within 45 cm in one day and harvest 6495 g seeds per hectare. Seeds collected from male sterile line formed the F1 hybrid, but half of them were fertile.

According to the inheritance of male sterility, a two-step method for hybrid seed production has been developed. The first is production of F1 hybrid and the second is asexual reproduction of male sterile plant roots. Given the fact that half of F1 hybrid seeds are fertile that will be identified till to flowering [3]. There are two asexual reproduction methods for male sterile plants except for tissue culture, cutting of stem base connecting to root and root cutting. Cutting of stem base has lower propagation coefficient (2–3) than root cutting (4–5), thus resulting in less application in production. When we obtained sufficient roots cuttings as male sterile “seeds”, F1 hybrid seeds can be produced in large scale.

Marker-assisted selection (MAS) of simple traits and quantitative traits is helpful to improve the efficiency of breeding programs for many crops [18]. To further use this male sterile system for hybrid seed production, it is very important to breed maternal parents. The problem of male sterility of *Salvia miltiorrhiza* was that the maternal parents are segregated to 50% male-fertile and 50% male-sterile plants in hybrid seeds. Therefore, the fertile should be identified and removed as early as possible before flowering stage. Development of a large-scale selection system combined with stable PCR-based assay will exclude all male-fertile plants at seedling stage before planting in the seed production field. If cheap, fast and reliable PCR-based markers were available for the male sterile gene, it would greatly improve the efficiency of breeding program. Consequently, conversion of a polymorphic AFLP fragment tightly linked to male sterile gene into a gene-specific marker is the next step for marker-assisted selection breeding in *Salvia miltiorrhiza*.

### Identification of AFLP Marker Linked to Male Sterile Gene

In AFLP analysis, the fertile and sterile DNA bulks were used to identify putative markers linked to male sterile gene. The assays involved two common enzymes (EcoRI and MseI). A total of 128 pairs of primer combinations were used with E+3/M+3. Fourteen primer combinations revealed polymorphism between the fertile and sterile DNA bulks (Fig. 2). Each primer combinations amplified fragments ranging from 25 to 120 with an average of 37.90. These fragments were widely dispersed and ranged in size from 100 to 1000 bp, mainly concentrating on ca. 500 bp.

Examination of eight fertile and eight sterile individuals in the bulks indicated that one of fourteen AFLP markers, E11/M4_208_ (E11: 5′-GACTGCGTACCAATTCACC-3′, M4: 5′-GATGAGTCCTGAGTAACAG-3′), was associated with male sterile gene (Fig. 3). This AFLP marker was confirmed in the 136 fertile and 137 sterile plants from F1 mapping population (*χ* ^2^-test 3.84, Probability 0.137) and then was cloned and sequenced. Electrophoresis results indicated that this marker segregated in sterile and fertile plants corresponding with the expected Mendelian ratio of 1∶1. Linkage analysis confirmed that this AFLP marker was tightly linked to male sterile gene with a recombination frequency of 6.85% and at a distance of 6.89 cM.

As mentioned above, half of fertile plants containing in F1 hybrid populations need to be removed from the field as early as possible, however, they were identified as far as flowering stage in nowadays. The identified E11/M4208 marker can easily be converted into sequence characterized amplified region (SCAR) marker, which is a fast, cheap and reliable PCR-based assay used to identify large amount of individuals in a target population. It will greatly facilitate the identification of fertile plants as early in seedling stage by using this SCAR marker and make the large-scale production of male sterile seedlings through seeds possible because those fertile ones can be identified and then removed before transplanted in field. Under this circumstance, large scale F1 hybrid seed production using sexual propagation was realized and thus reduced the great deal of labor cost in cutting root.

### Sequence Features of Male Sterile Gene Amplified by E11/M4 Primer Combination

The fragment (208 bp) amplified by E11/M4 primer combination was submitted to the NCBI website (http://www.ncbi.nlm.nih.gov/) for nucleotide-nucleotide BLAST (Blastn) analysis. The sequence was identical to nucleotides in *Arabidopsis* genome ranging from 84 to 100% and in *Oryza sativa* japonica genome with the percentage of 82–100% (Table 3).

Both phosphoethanolamine N-methyltransferase (PEAMT) and choline/ethanolamine phosphotransferase play key roles in phosphatidylcholine (Ptd-Cho) and glycinebetaine (GlyBet)biosynthesis [19]. The PEAMT gene was found highly expressing in *Salicornia europaea* pollen, root, petiole and leaf, which activity was induced by low temperature, abscisic acid (ABA) and NaCl [20]. MADS-box protein was also found highly expressing in flower bud of cytoplasmic male sterile soybean (*Glycine max* L. Merr.) plants [21].

Zeaxanthin epoxidase (ZEP) plays an important role in ABA biosynthesis and participates xanthophyll cycle in higher plants, which can catalyze zeaxanthin into anthraxanthin and violaxanthin by using excessive light energy and confirm photosynthesis can be smoothly proceeded under lower light [22]. The anthraxanthin is the expansion section of stamen that producing pollens, which function would affect the activity of pollens.

S-adenosylmethionine (AdoMet) is a common co-substrate involved in methyl group transfers. Transmethylation, transsulfuration, and aminopropylation are the metabolic pathways that use AdoMet. On the other hand, DNA methylation involves the development of plant gametogenesis [23]. Depletion of *A*. *thaliana* MET1 results in immense epigenetic diversification of gametes and this diversity seems to be a consequence of passive postmeiotic demethylation, leading to gametes with fully demethylated and hemidemethylated DNA [24].
